# Supplementary material for: Integrated single-cell atlas of human atherosclerotic plaques
Source: Nat Commun. 2025 Sep 10;16:8255. doi: 10.1038/s41467-025-63202-x (PMC12423310; doi:10.1038/s41467-025-63202-x)
Supplement: Supplementary file 1 — Supplementary Information [file 41467_2025_63202_MOESM1_ESM.pdf]

Supplementary Material

Integrated single-cell atlas of human atherosclerotic plaques

K. Traeuble, M. Munz, J. Pauli, N. Sachs, E. Vafadarnejad, T. Carrillo-Roa, L. Maegdefessel, P. Kastner, M. Heinig

Supplementary Figures

Pan sample 2

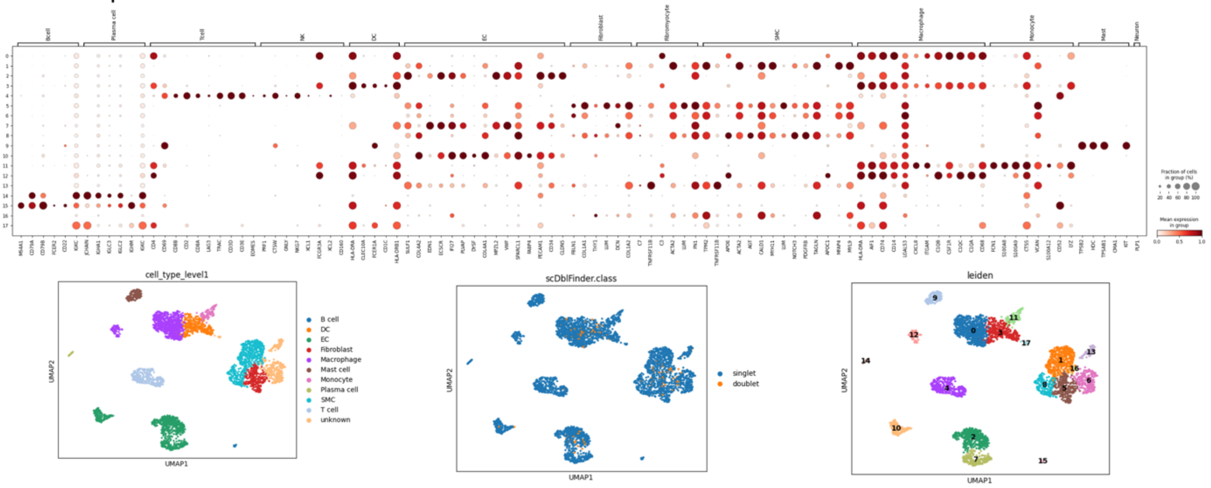

Pan sample 3

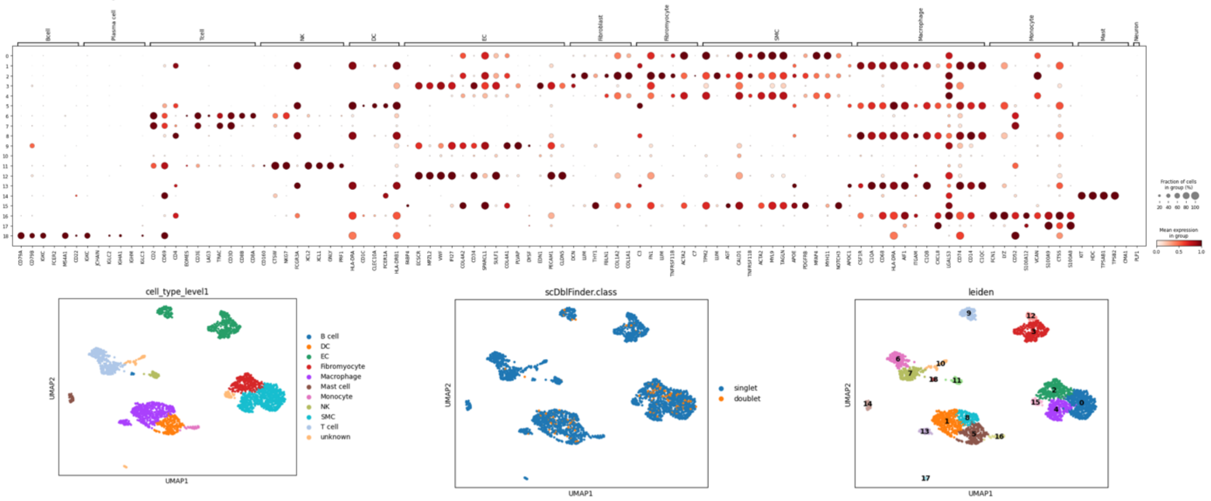

Slysz sample 6

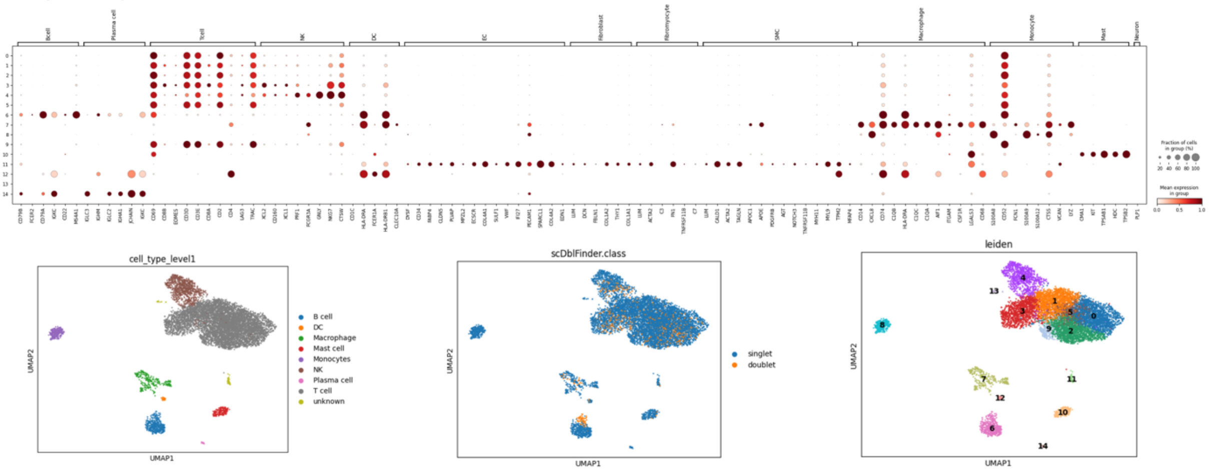

Slysz sample 8

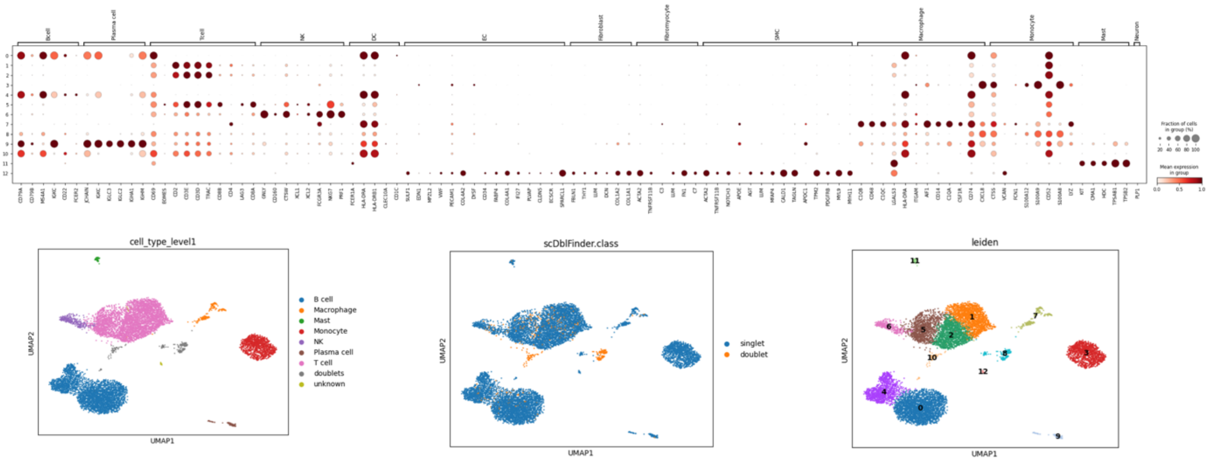

Wirka sample 5

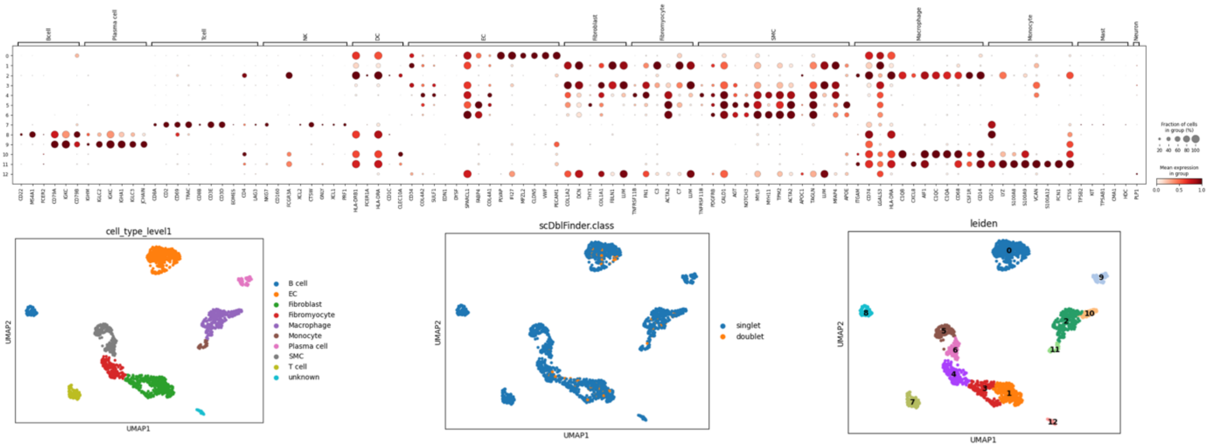

Wirka sample 6

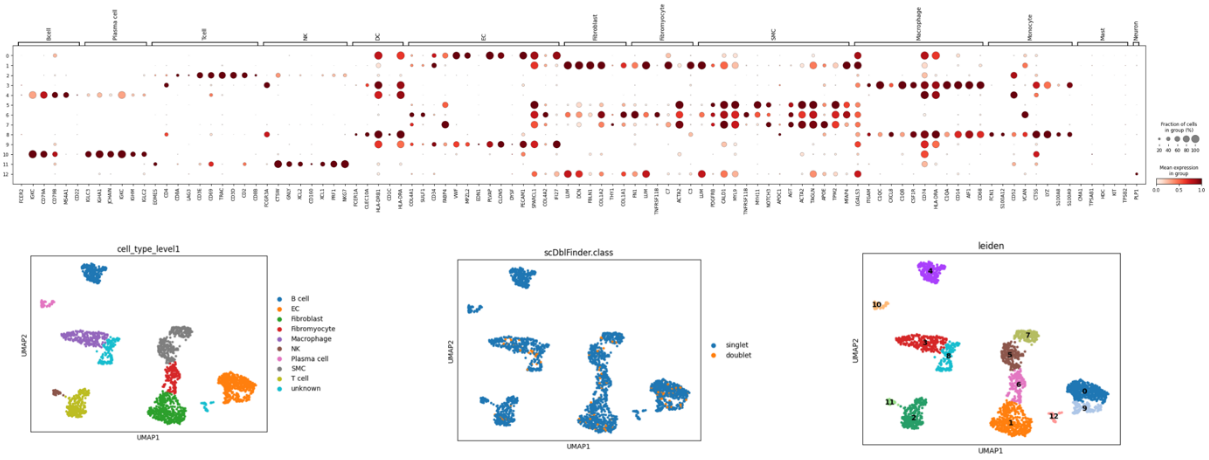

Wirka sample 7

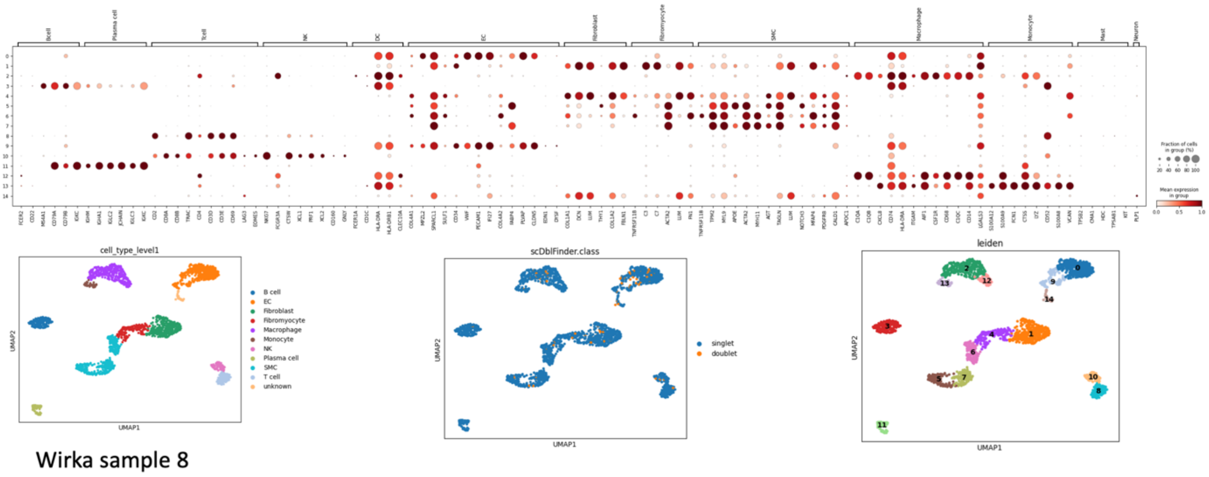

Wirka sample 8

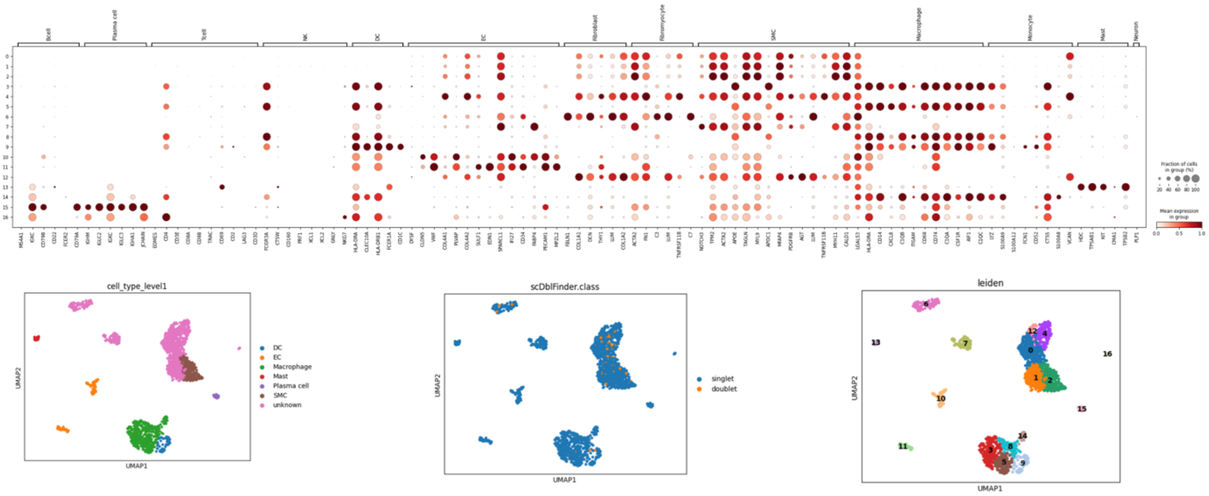

Alsaigh sample 3

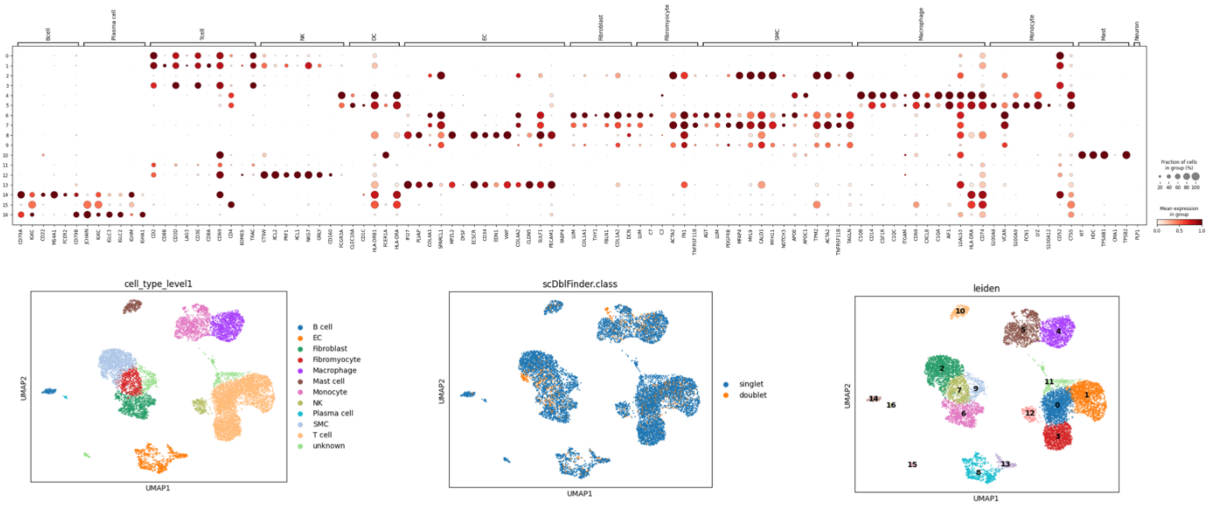

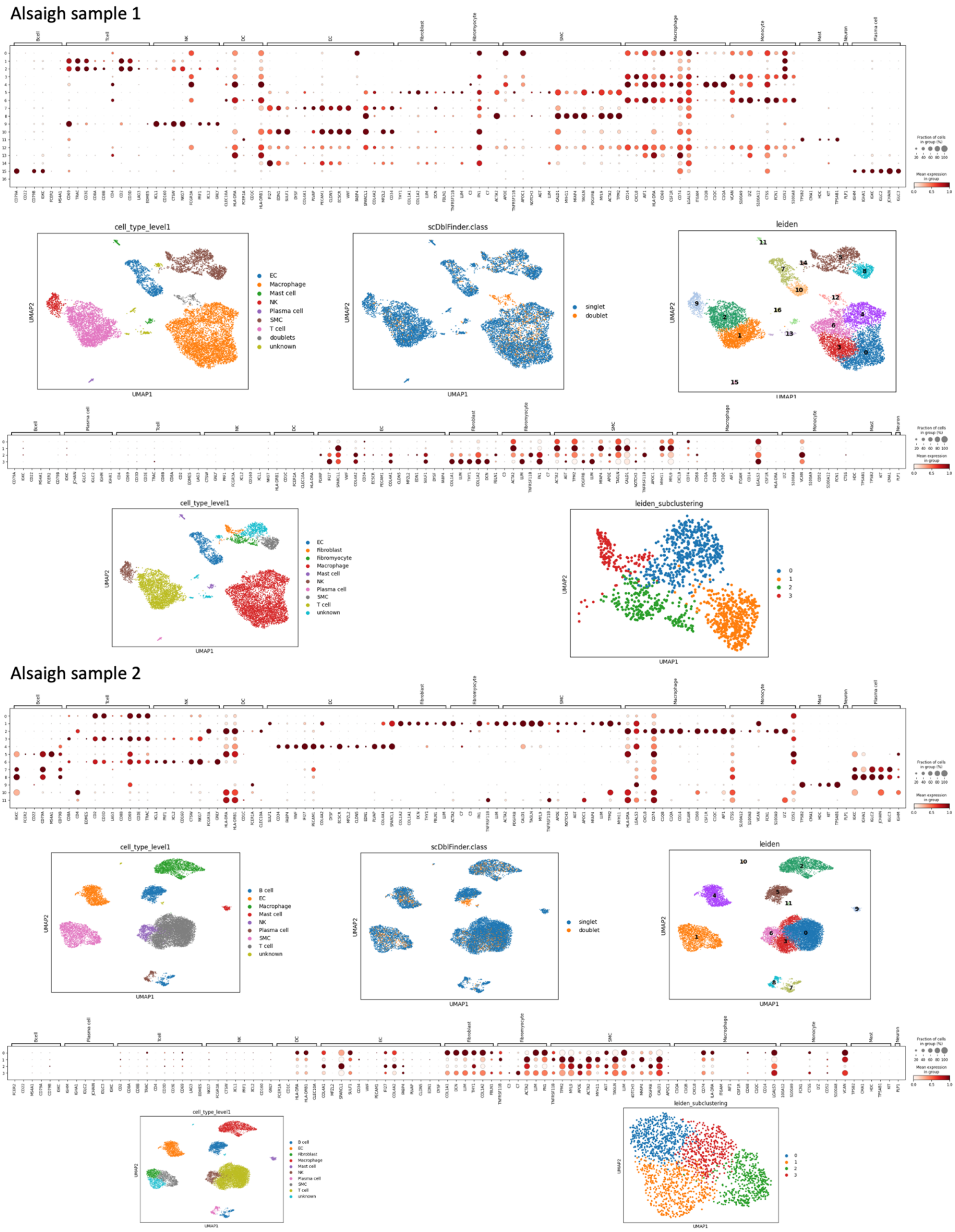

Suppl. Figure 1*: Manual annotation of a subset of the samples in the atlas using marker genes.* For the manual annotation several samples were preprocessed and annotated with dedicated marker genes. For each sample the dot plot with the Leiden clusters on the y-axis and the marker genes on the x-axis is shown, together with the corresponding UMAPs of the annotated cells with Level 1, the doublet classification from ScDblFinder, and the Leiden clusters. In some cases, sub-clustering on specific clusters were performed and the corresponding dot plot, cell type UMAP and Leiden UMAP shown.

Method	Bio conservation					Batch correction					Aggregate score		
	Isolated labels	KMeans NMI	KMeans ARI	Silhouette label	cLISI	Silhouette batch	iLISI	KBET	Graph connectivity	PCR comparison	Batch correction	Bio conservation	Total
scPoli	0.64	1.00	1.00	1.00	1.00	0.84	0.64	0.54	0.89	0.16	0.61	0.93	0.80
scPoli_nb	0.22	1.00	0.82	0.99	1.00	0.87	0.91	0.99	0.80	0.14	0.74	0.80	0.78
Harmony	1.00	0.79	0.84	0.40	1.00	0.94	0.60	0.33	0.59	0.48	0.59	0.81	0.72
scGen	0.37	0.80	0.52	0.43	1.00	1.00	0.60	0.37	1.00	0.84	0.76	0.62	0.68
scANVI	0.51	0.77	0.91	0.37	1.00	0.63	0.54	0.29	0.82	0.72	0.60	0.71	0.67
PCA	0.87	0.67	0.53	0.24	1.00	0.04	0.00	0.00	0.61	0.00	0.13	0.66	0.45
scVI	0.32	0.00	0.00	0.00	0.65	0.80	0.47	0.11	0.79	0.99	0.63	0.19	0.37
LIGER	0.00	0.29	0.42	0.20	0.00	0.00	1.00	1.00	0.00	1.00	0.60	0.18	0.35

Suppl. Figure 2: *Benchmark of integration methods with scib-metrics on a subset of manually annotated samples.* For each method, ten metrics are calculated and normalized between 0 and 1 across the methods. Five Bio conservation and five batch correction metrics are aggregated in one total score. scPoli was the best performing method in bio conservation and in total. Source data are provided as a Source Data file.

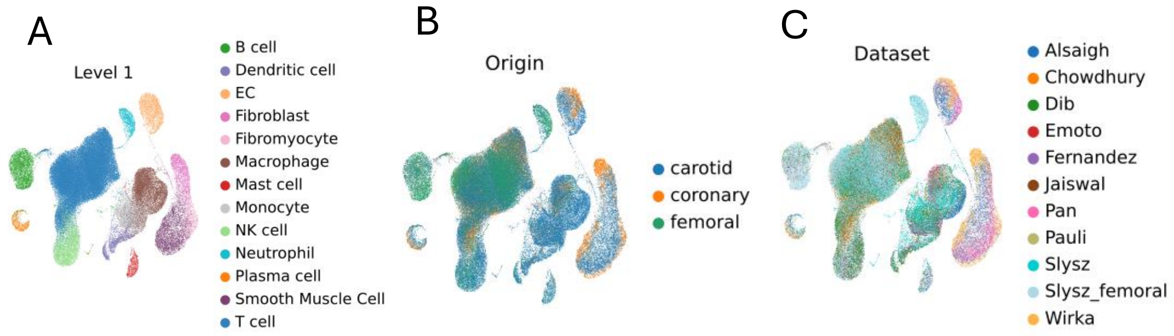

Suppl. Figure 3*: Reference atlas after level 1 integration.* A) UMAP of the embedding after integration including Level 1 annotations. B) UMAP with the origin site of the cells tissue. C) UMAP with the different datasets used in the reference atlas.

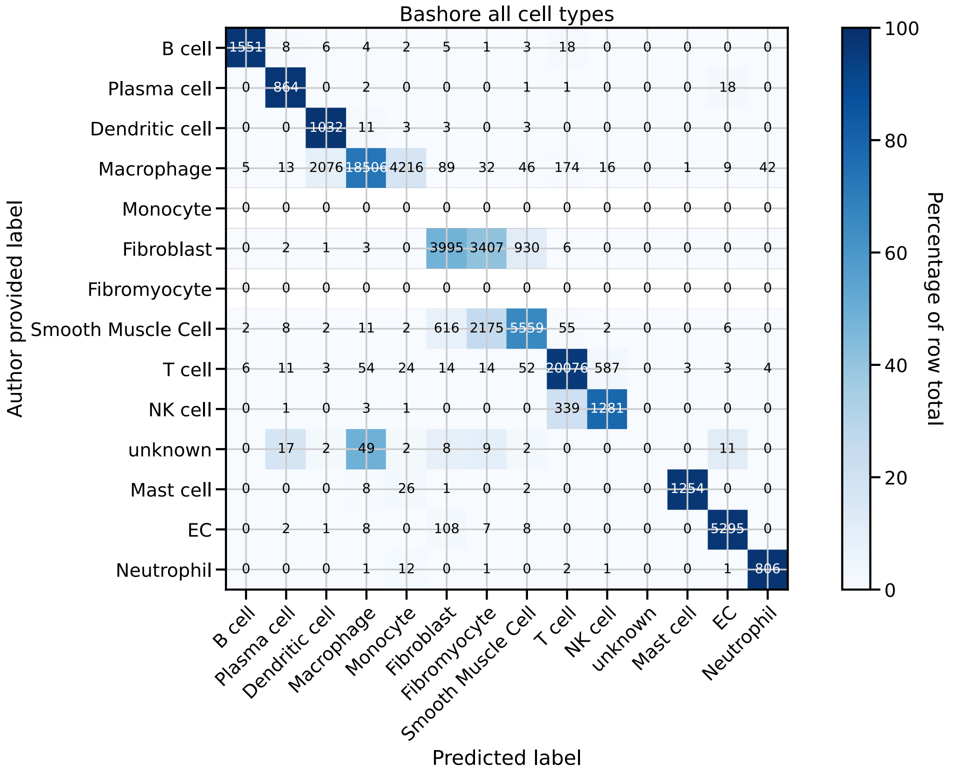

Suppl. Figure 4: *Validation of the cell type label transfer.* Confusion matrix of the Bashore et al. mappings with all cell types, including the cell types that are not shared between the author provided labels and our predicted labels. To compensate for the differences in cell type abundance the colormap is logarithmized and the overall precision and recall are weighted according to their abundance. Source data are provided as a Source Data file.

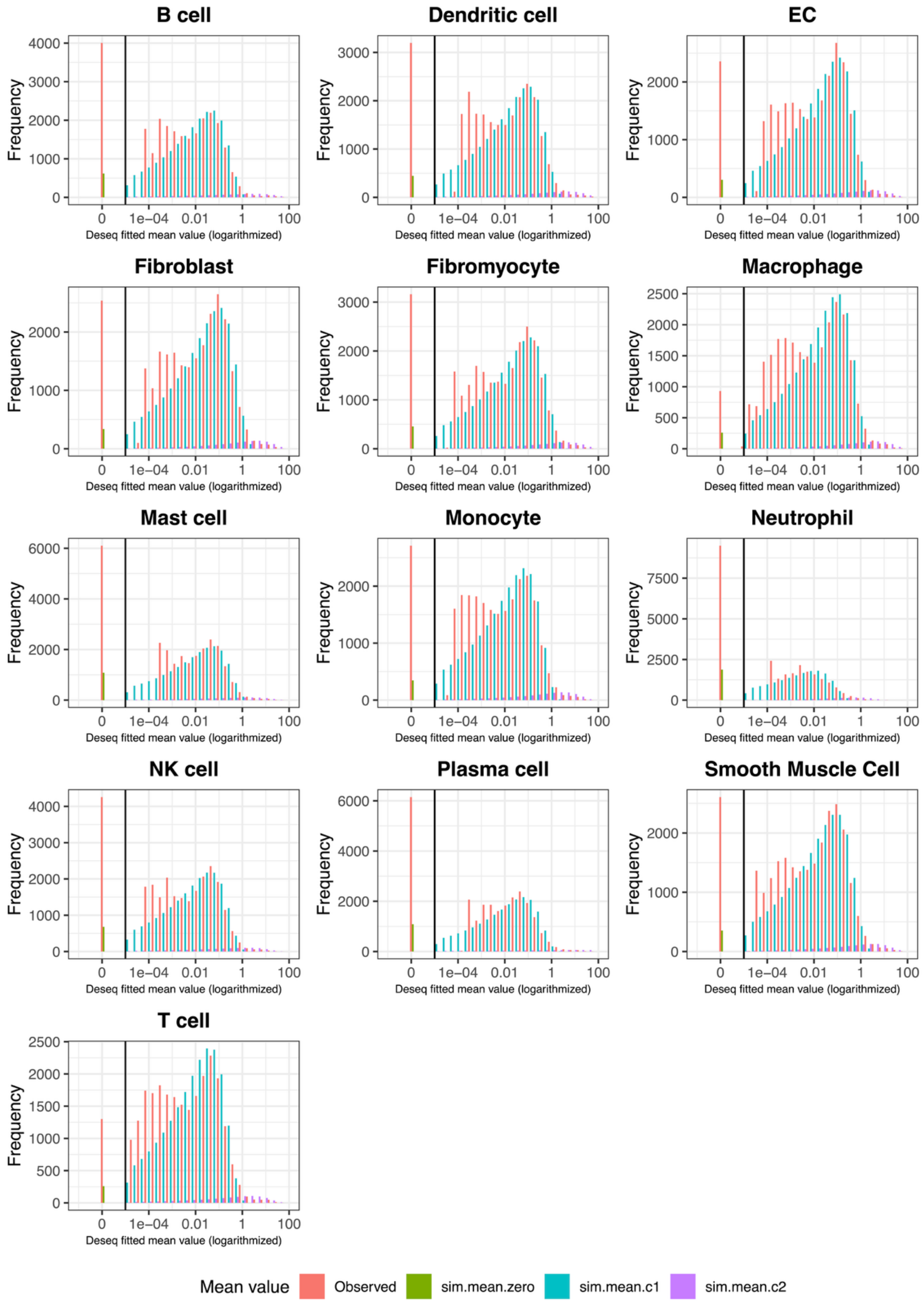

Suppl. Figure 5*: Gene expression gamma prior fits used for scPower for each cell type using the atlas as a reference.* The panels depict the frequency of the Deseq fitted mean values (logarithmized) stratified in the zero component, two gamma distributions and the observed values per cell type. Source data are provided as a Source Data file.

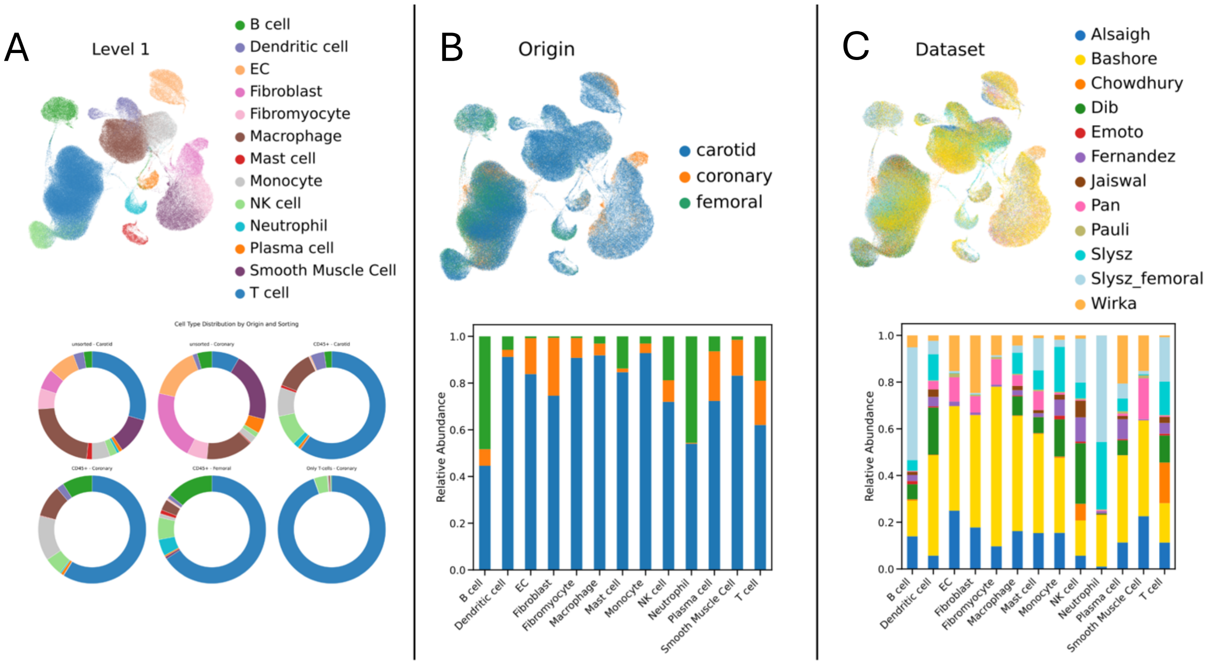

Suppl. Figure 6*: Abundances of cells per sorting and per cell type, origin and dataset.* A) UMAP and cell type abundances by origin and sorting. B) UMAP and origin abundance of cells by cell type. C) UMAP and dataset abundance per cell type. Source data are provided as a Source Data file.

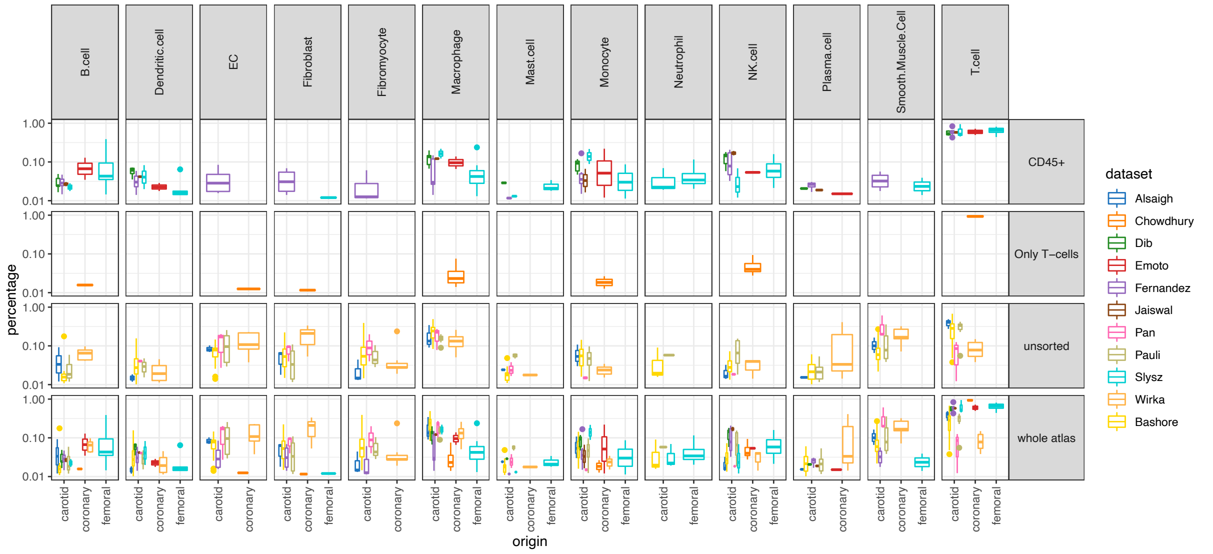

Suppl. Figure 7: *Abundance comparisons by origin and sorting in the atlas.* Absolute cell type abundances per sample were converted into percentages, with values below 1% excluded. The y-axis was log-transformed to highlight differences in smaller abundances. Comparisons were stratified by cell type, origin, and sorting method. The final row summarizes abundances across all sorting algorithms. In the legend, the *Slysz_femoral* and *Slysz* datasets are pooled, as they are differentiated by origin in the plot. Source data are provided as a Source Data file.

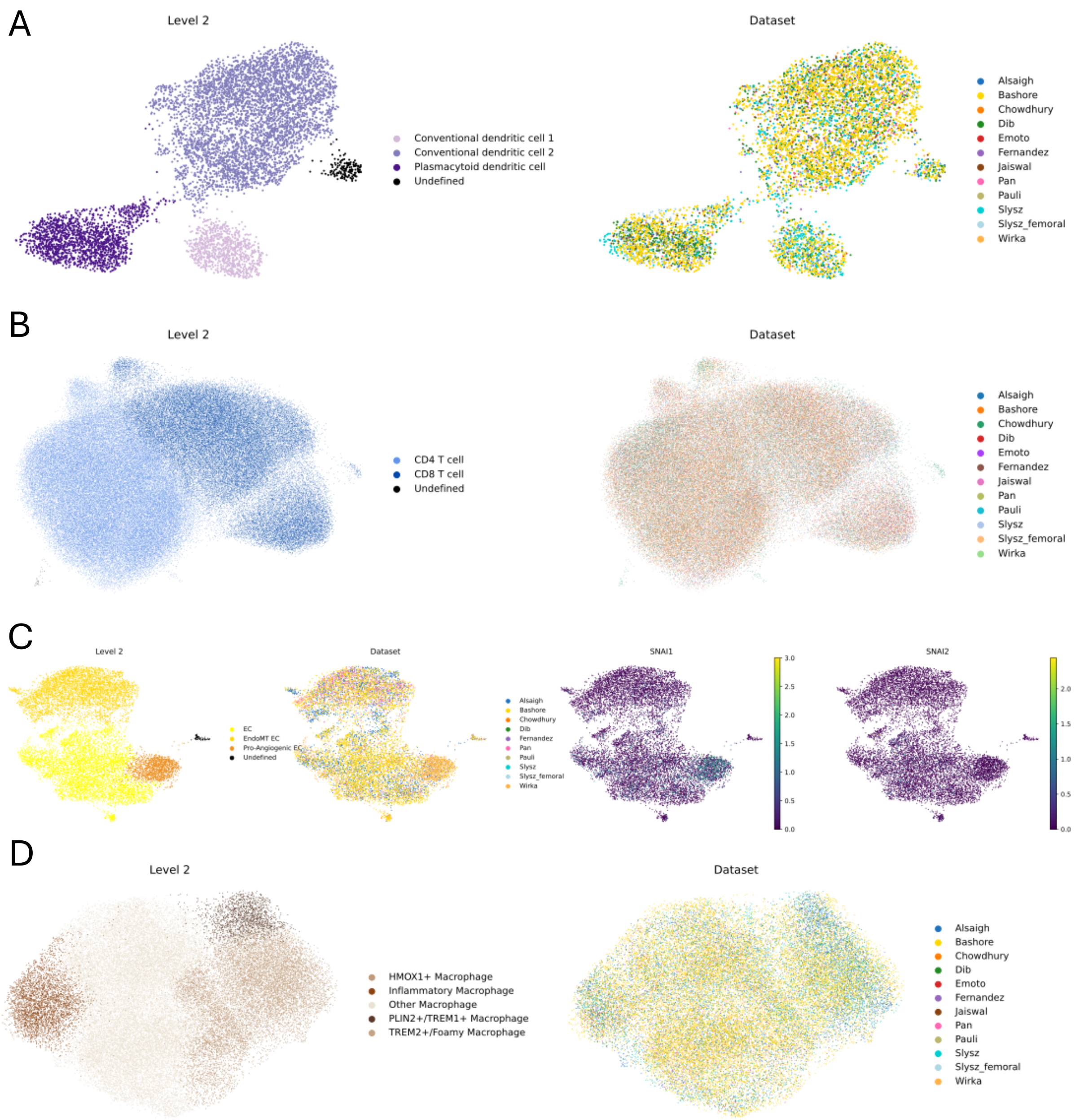

Suppl. Figure 8: *Level 2 annotation of subclusters.* UMAPs for the level 2 sub clustering for A) Dendritic cells, B) T cells, C) Endothelial cells, D) Macrophages. Cells with no clear signature were termed Undefined.

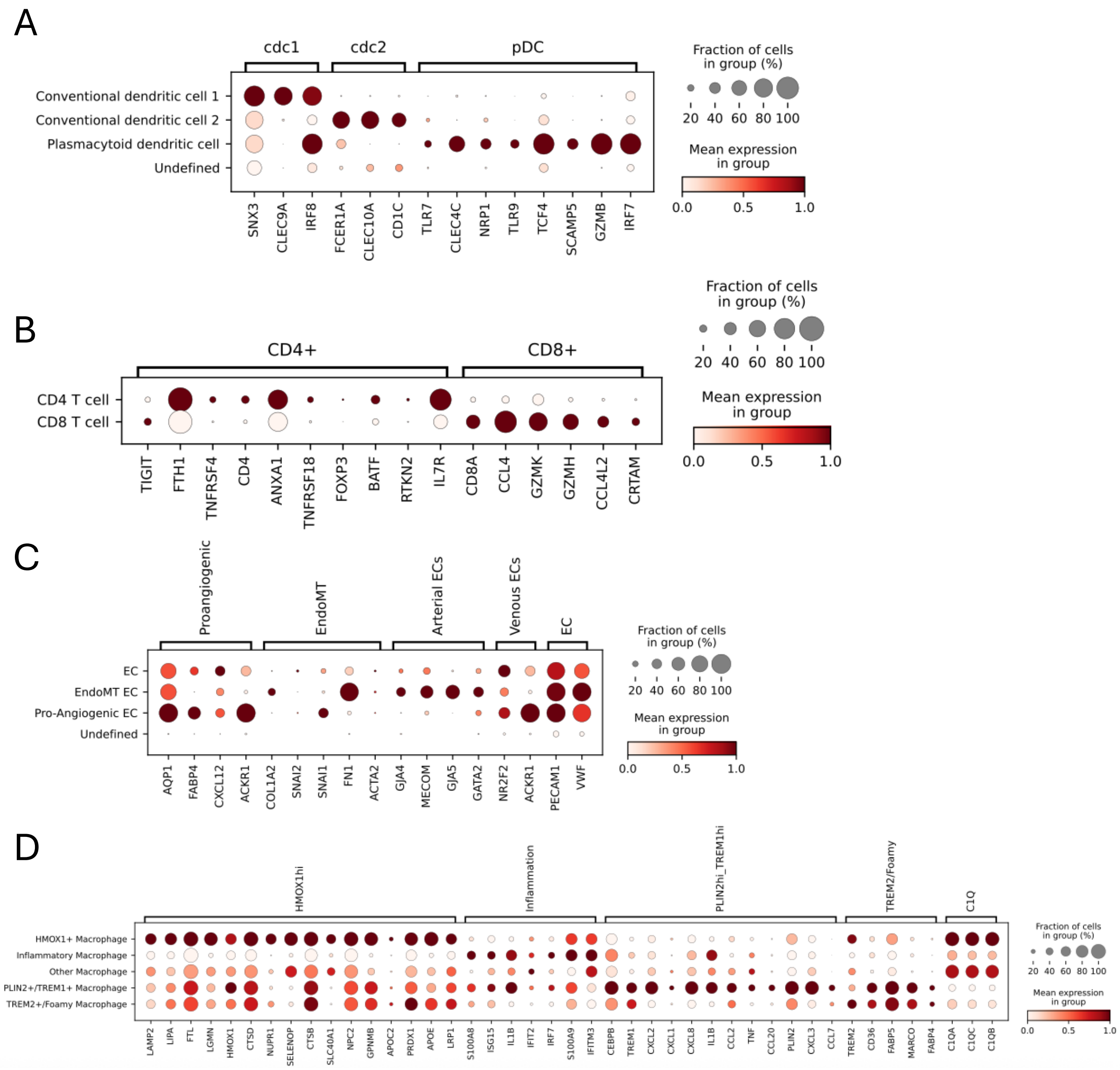

Suppl. Figure 9: *Level 2 annotation of subclusters.* Dot plots for the level 2 sub clustering for A) Dendritic cells, B) T cells, C) Endothelial cells, D) Macrophages.

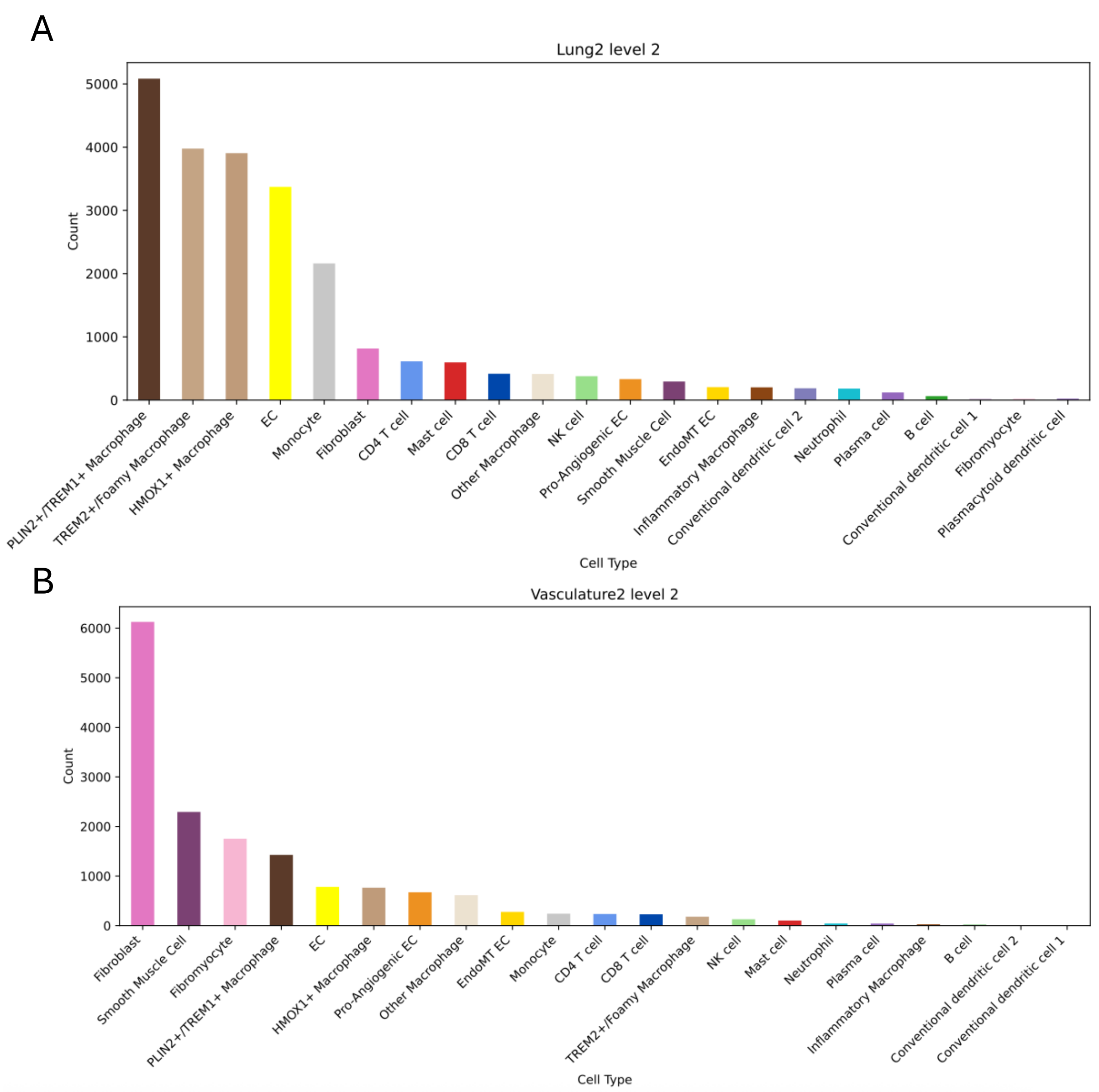

Suppl. Figure 10*: Abundance of the predicted level 2 cell types in Tabula Sapiens.* Level 2 cell type abundance of the Tabula Sapiens subsets after mapping the Lung (A) and Vasculature (B) dataset to the plaque atlas. Source data are provided as a Source Data file.

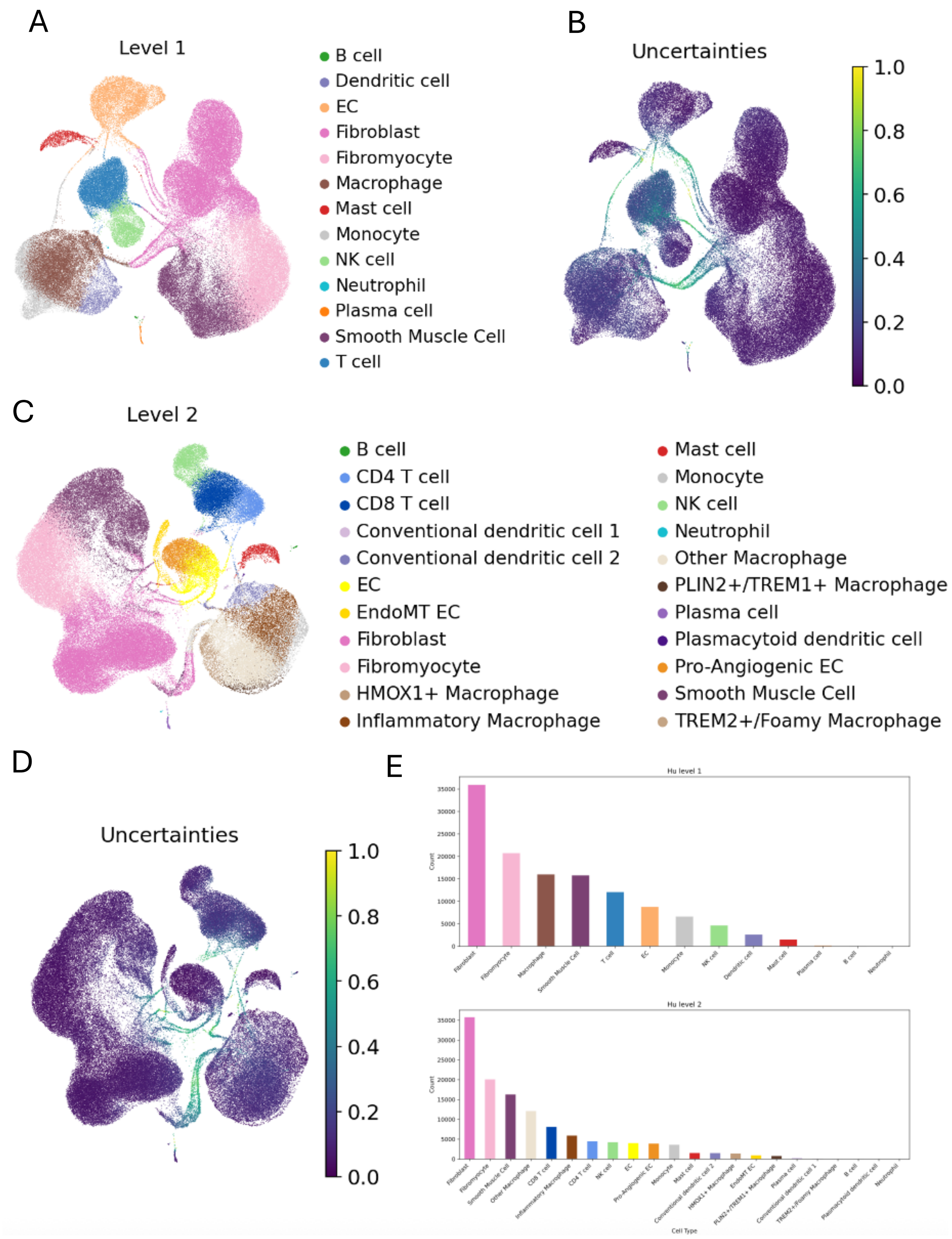

Suppl. Figure 11: *Reference mapping of healthy arteries of the Hu et al. dataset on the plaque atlas.* UMAPs of the mapped Hu et al. cells to the plaque atlas and their uncertainties depicted with Level 1 (A+B), Level 2 (C+D) and the level 1 and level 2 cell type abundance after excluding cells with a higher uncertainty then 0.7 (E). Source data are provided as a Source Data file.

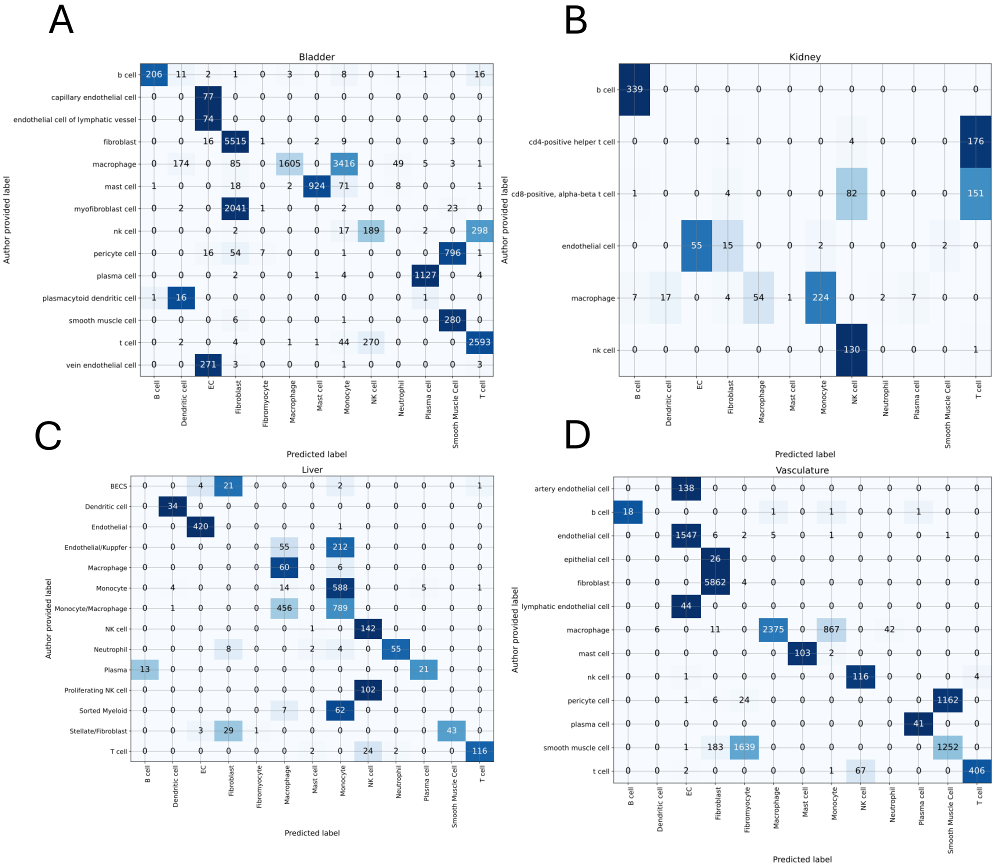

Suppl. Figure 12: *Confusion matrices for the Tabula sapiens mappings.* Panels A-D show the confusion matrices for different data sets (data set name given in the panel headers) providing the number of cells with specific combinations of expert annotations (“Author provided label” on the y-axis) and labels predicted by the atlas (“Predicted Label” on the x-axis). Organ specific cell types and cells with a higher uncertainty than 0.7 are removed. The colormap is normalized for each row to compensate for differences in cell type abundance. Source data are provided as a Source Data file.

Supplementary Tables

**Level 1**	**Level 2**	**Marker genes**	**Role in disease progression**
B cell	B cell	CD79A[1–5], CD79B[3–5], MS4A1[1], IGKC[1], CD22[4,6], FCER2[4]	Plaque formation regulation through production of antibodies and cytokines[7]
Mast cell	Mast cell	TPSAB1[8,9], TPSB2[2,4,8], KIT[4,10,11], HDC[4,11], CMA1[4,11]	Can influence plaque stability under stress in mouse model[12]
T cells	T cell	CD2[1], TRAC[1], CD69[1], CD3E[13], CD3D[2], CD4[2], CD8A[2], CD8B[2], EOMES[14], LAG3[14]	T cells are the predominant lymphoid cells in atherosclerotic plaques, where they are more activated, differentiated, and exhausted compared to their blood counterparts, indicating a significant role in disease progression[14,15]
T cell	CD4	IL7R[15], ANXA1[15], FTH1[15], CD4[14,15], BATF[15], TNFRSF4[15], TNFRSF18[15]	An expansion of activated effector memory CD4+ T cells, particularly in symptomatic patients, suggests their involvement in promoting inflammation and contributing to atherosclerosis progression following recent cardiovascular events[14,15]
T cell	CD8	CCL4L2[15], CRTAM[15], GZMK[15], CCL4[15], CD8A[15], GZMH[15]	Cytotoxic CD8+ T cells are more abundant in carotid plaques compared to femoral plaques; they

			exhibit activation, differentiation, and exhaustion signatures, indicating their significant role in promoting inflammation and cytotoxicity in atherosclerosis[14,15]
Fibroblast	Fibroblast	LUM[3,8], DCN[3,8], COL1A1[8], COL1A2[8], FBLN1[8], THY1[2], C3[3], C7[3]	Fibroblasts play a role in all stages of atherosclerosis including initial phase, fibrous cap formation and plaque formation. Functional and phenotypic changes yield a high diversity and plasticity in their role in the disease[16]
Natural killer cell	Natural killer cell	NKG7[1], XCL1[1,17], CTSW[1], XCL2[17], CD160[17], FCGR3A[17], PRF1[17], GNLY[17]	In human atherosclerosis, their role is still not completely known. One study suggests a role in plaque stability[18]
Endothelial cell	Endothelial cell	PECAM1[1,3,4,8], VWF[1,2,4,8], FABP4[8], CLDN5[3,8], IFI27[8], ECSCR[1], DYSF[2], CD34[4], COL4A1[4], COL4A2[4], SPARCL1[4], PLVAP[4], MPZL2[4], SULF1[4], EDN1[4]	Endothelial cells play a central role in the development of atherosclerosis by becoming dysfunctional and inflamed in response to risk factors, especially in areas of disturbed blood flow, which leads to monocyte recruitment, LDL oxidation, foam cell formation, and a cycle of chronic inflammation[19]
Endothelial cell	Proangiogenic	ACKR1[3], AQP1[3,20],	Proangiogenic

		FABP4[3,21], CXCL12	phenotype which promotes angiogenesis, vascular permeability and leukocyte recruitment[22]
Endothelial cell	EndoMT	COL1A2[3], FN1[3]	ECs that acquire a myofibroblast-like phenotype and lose their EC characteristics. This process is important during arterial and cardiovascular remodeling, which may affect plaque stability[23]
Smooth muscle cell	Smooth muscle cell	ACTA2[4,8], MYH11[2,4,8,24], MYL9[1,8], TPM2[8], CALD1[1], TAGLN[1,4], TNFRSF11B[2], LUM[2], APOE[3], APOC1[3], AGT[3], NOTCH3[4,25], PDGFRB[4,25], MFAP4[4,26]	Smooth muscle cells play dual roles in atherosclerosis by contributing to lesion expansion and forming a stabilizing fibrous cap, though they also undergo phenotypic switching to macrophage-like, foam cell-like, and other forms that influence plaque growth and stability, potentially increasing plaque vulnerability[27]
Fibromyocyte	Fibromyocyte	FN1[8,28], LUM[28], TNFRSF11B[28], ACTA2[3], TCF21[28]	Modulated SMCs that transform into fibroblast-like cells, that establish a protective role in coronary artery disease[28]
Dendritic cell	Dendritic cell	CLEC10A[3,4,9,10], FCER1A[4,9,10], CD1C[3,4,10,29], HLA-DRA[10], HLA-DRB1[10]	They act as antigen-presenting cells in atherosclerosis, driving T-cell responses and

			inflammation that advance disease progression[30]
Dendritic cell	cDC1	CLEC9A[17], IRF8[17], SNX3[17]	Conventional dendritic cell subtype 1 (cDC1) may help stabilize plaques by promoting atheroprotective Treg cells[30]
Dendritic cell	cDC2	CD1C[17], CLEC10A[17], FCER1A[17]	Conventional dendritic cell subtype 2 (cDC2) supports inflammation by recruiting pro-atherogenic T helper cells[30]
Dendritic cell	Plasmacytoid dendritic cell	TCF4, GZMB, TLR7, TLR9, NRP1, SCAMP5, CLEC4C, IRF7. Marker genes are taken from Ghanem et al[31].	Plasmacytoid dendritic cells (pDCs) contribute to vascular inflammation and atherogenesis by producing type I interferons (IFN-I) and chemokines, which recruit inflammatory cells to sites of inflammation[32]
Neutrophil	Neutrophil	NAMPT[33], IFITM2[34], G0S2[35], CXCL8[36], NEAT1[35], SRGN[37], AQP9[38], SOD2, FCGR3B, IVNS1ABP	Neutrophils are rarely found in atherosclerotic lesions but can promote LDL vascular deposition and oxidation during early stages of atherosclerosis[39]
Plasma cell	Plasma cell	IGKC[8], IGHM[3,8], IGHA1[8], IGLC2[3,8], IGLC3[8], JCHAIN[3]	Produce antibodies and can regulate plaque formation[7]
Monocyte	Monocyte	FCN1[4], S100A8[3,4,10], S100A9[3,4,10], S100A12[4,10], VCAN[4],	Monocytes infiltrate the intima and differentiate into

		CD52[4], LYZ[3,10], CTSS[10]	macrophages that influence plaque stability[40]
Macrophage	Macrophage	C1QA[8], C1QB[8], C1QC[8], CD74[8,14,41], CXCL8[8], AIF1[1], CD14[1,4,10], CD68[1,4,10], ITGAM[10], CSF1R[10], HLA-DRA[14], LGALS3[4]	Macrophages play several roles in atherosclerosis depending on the subset[40]
Macrophage	Foamy	TREM2[4,42–45], MARCO[17], FABP4/5[17], CD36[17].	Foam cells are the hallmark of atherosclerosis. They take up modified LDL cholesterol and form the plaque[40]
Macrophage	Inflammatory	S100A8[17], IL1B[17], S100A9[17],IRF7[46], IFITM3[46], ISG15[46], IFIT2[46]	These macrophages promote inflammation in plaques and hence participate substantially in plaque destabilization and rupture[40]. However this sub cell types role is under active discussion depending on the definition.
Macrophage	Other	No distinct markers within macrophages	Macrophages with no indicative signature.
Macrophage	PLIN2/TREM1	PLIN2, TREM1, CXCL1/2/3/8, CCL2/7/20, IL1B, TNF, CEBPB. All marker genes are taken from Dib et al[17].	They are introduced by Dib et al[17] and their signature is correlated with vascular events. However, our results indicate no active role with respect to abundance of this cell type.
Macrophage	HMOX1+	HMOX1, APOC2, GPNMB, LIPA, NPC2, PRDX1, SLC40A1, NUPR1,	This sub cell types role is still not fully defined.

		APOE, LAMP2, CTSB, SELENOP, LGMN, LRP1, CTSD, FTL. All marker genes are taken from Dib et al[17].	

Suppl. Table 1: *Summary of cell types included in the atlas*. The first column presents the Level 1 annotation, while the second column provides the corresponding finer-grained Level 2 annotation. The third column lists marker genes along with their associated references. The final column outlines the role of each cell type in disease progression.

cell_type	t_stat	df	p_value	p_adj	ci_lower	ci_upper	label
NK cell	-5.432763738	108.2334228	0.0000003447322452	0.000002240759594	-3.779801166	-1.758991737	t(108.2) = -5.433, p = 3.4e-07
Plasma cell	-5.106035325	112.1992516	0.000001357275007	0.000005881525032	-4.062727499	-1.791188377	t(112.2) = -5.106, p = 1.4e-06
T cell	-4.181707942	117.8230903	0.00005590930092	0.0001211368186	-3.231860513	-1.154596048	t(117.8) = -4.182, p = 5.6e-05
Mast cell	-6.256553144	71.52004444	0.00000002566042161	0.000000333585481	-3.850528417	-1.989549016	t(71.5) = -6.257, p = 2.6e-08
Monocyte	-4.244020051	123.2951268	0.00004276903439	0.0001111994894	-3.795024825	-1.380966194	t(123.3) = -4.244, p = 4.3e-05
Dendritic cell	0.2174560468	111.2911931	0.8282511355	0.8282511355	-1.009458401	1.258331605	ns
Neutrophil	-1.428966512	99.56797923	0.1561436692	0.1714371415	-2.006490374	0.3263432601	ns
B cell	-3.061233516	98.68370664	0.002839856312	0.003691813206	-3.817726317	-0.8148707496	t(98.7) = -3.061, p = 2.8e-03
Smooth Muscle Cell	3.764370466	101.1518905	0.0002803723395	0.0004556050517	1.230701808	3.972736265	t(101.2) = 3.764, p = 2.8e-04
EC	-1.42875124	60.13491828	0.1582496691	0.1714371415	-0.753483416	0.1255721064	ns
Fibroblast	-4.470313289	68.73699793	0.00003000279226	0.00009750907484	-4.54371826	-1.739531091	t(68.7) = -4.470, p = 3.0e-05
Macrophage	-3.952417962	61.84782884	0.0002013439021	0.0003739243895	-1.550020635	-0.5087351252	t(61.8) = -3.952, p = 2.0e-04
Fibromyocyte	3.155359873	184.8884231	0.001871588996	0.002703406328	0.244994364	1.06250178	t(184.9) = 3.155, p = 1.9e-03

cell_type	t_stat	df	p_value	p_adj	ci_lower	ci_upper	label
Conventional dendritic cell 2	0.2585641005	96.5123014	0.7965222091	0.8344518381	-0.7441109903	0.9670177584	ns

CD8 T cell	-2.32254349	124.1085059	0.02183208483	0.03430756187	-1.670923147	-0.1333382148	t(124.1) = -2.32254, p = 2.2e-02
NK cell	-6.233632534	112.9751211	0.000000008089978863	0.00000008898976749	-4.027395093	-2.084804168	t(113) = -6.23363, p = 8.1e-09
Plasmacytoid dendritic cell	-0.3245483386	111.0980121	0.7461332236	0.8344518381	-1.13276463	0.813925427	ns
Plasma cell	-4.184633609	113.3320527	0.00005662016091	0.0001557054425	-3.528061998	-1.260858752	t(113.3) = -4.18463, p = 5.7e-05
Monocyte	-4.921325594	146.3851864	0.000002287318527	0.0000125802519	-3.277689937	-1.39944831	t(146.4) = -4.92133, p = 2.3e-06
Inflammatory Macrophage	-0.001691844058	108.9901371	0.9986531972	0.9986531972	-1.038725067	1.036953231	ns
Neutrophil	-3.400719583	99.03347275	0.0009702913276	0.001940582655	-3.024288831	-0.7955422966	t(99) = -3.40072, p = 9.7e-04
Mast cell	-6.009763824	70.42344898	0.00000007403204001	0.0000005429016268	-3.595874829	-1.804021507	t(70.4) = -6.00976, p = 7.4e-08
Conventional dendritic cell 1	-2.123672624	105.2922537	0.0360397572	0.05285831056	-2.856775482	-0.09803690661	ns
CD4 T cell	0.2957487534	104.701222	0.7680077136	0.8344518381	-0.9026848067	1.21916014	ns
B cell	-3.685887921	96.95960016	0.0003756077879	0.0008263371335	-4.239746271	-1.271899826	t(97) = -3.68589, p = 3.8e-04
EndoMT EC	6.861169339	119.6985861	0.0000000003216009964	0.000000007075221921	2.768341814	5.014214963	t(119.7) = 6.86117, p = 3.2e-10
Pro-Angiogenic EC	-2.91359114	107.5932988	0.004346393285	0.007355434791	-3.911495782	-0.7440801033	t(107.6) = -2.91359, p = 4.3e-03
PLIN2+/TREM1+ Macrophage	1.94121149	97.89054178	0.05510841945	0.07577407675	-0.03530326997	3.201904829	ns
Other Macrophage	1.188718152	121.9167921	0.2368608604	0.2894966072	-0.5358987541	2.1468139	ns
EC	-1.910021119	96.03121307	0.05911452365	0.07650114826	-2.759571524	0.05310493393	ns
TREM2+/Foamy Macrophage	-4.800843786	80.51904317	0.000007175243419	0.00003157107105	-5.34688767	-2.213314826	t(80.5) = -4.80084, p = 7.2e-06
Smooth Muscle Cell	4.274447503	102.2947466	0.00004309694474	0.0001354475406	1.569993989	4.288404826	t(102.3) = 4.27445, p = 4.3e-05
Fibroblast	-4.490331958	69.41020305	0.00002759236902	0.0001011720197	-4.505861312	-1.733954823	t(69.4) = -4.49033, p = 2.8e-05
HMOX1+ Macrophage	-4.028171879	70.09682403	0.0001406420769	0.0003437917434	-3.870006447	-1.306878365	t(70.1) = -4.02817, p = 1.4e-04
Fibromyocyte	3.080028636	191.1599506	0.002374835622	0.004353865307	0.215264741	0.9819819794	t(191.2) = 3.08003, p = 2.4e-03

Suppl. Table 2: *Summary test statistics*. Test statistics from level 1 and level 2 cell type deconvolution used in abundance comparisons of the bulk data.

References

1. Alsaigh, T., Evans, D., Frankel, D. & Torkamani, A. Decoding the transcriptome of calcified atherosclerotic plaque at single-cell resolution. *Commun. Biol.* **5**, 1084 (2022).
2. Wang, J. *et al.* Identification of immune cell infiltration and diagnostic biomarkers in unstable atherosclerotic plaques by integrated bioinformatics analysis and machine learning. *Front. Immunol.* **13**, 956078 (2022).
3. Mosquera, J. V. *et al.* Integrative single-cell meta-analysis reveals disease-relevant vascular cell states and markers in human atherosclerosis. *Cell Rep.* **42**, 113380 (2023).
4. Depuydt, M. A. C. *et al.* Microanatomy of the Human Atherosclerotic Plaque by Single-Cell Transcriptomics. *Circ. Res.* **127**, 1437–1455 (2020).
5. Luger, D. *et al.* Expression of the B-Cell Receptor Component CD79a on Immature Myeloid Cells Contributes to Their Tumor Promoting Effects. *PLoS ONE* **8**, e76115 (2013).
6. Tedder, T. F., Tuscano, J., Sato, S. & Kehrl, J. H. CD22, A B LYMPHOCYTE–SPECIFIC ADHESION MOLECULE THAT REGULATES ANTIGEN RECEPTOR SIGNALING [*]. *Annu. Rev. Immunol.* **15**, 481–504 (1997).
7. Pattarabanjird, T., Li, C. & McNamara, C. B Cells in Atherosclerosis. *JACC Basic Transl. Sci.* **6**, 546–563 (2021).
8. Shao, X. *et al.* Integrated single-cell RNA-seq analysis reveals the vital cell types and dynamic development signature of atherosclerosis. *Front. Physiol.* **14**, 1118239 (2023).
9. Villani, A.-C. *et al.* Single-cell RNA-seq reveals new types of human blood dendritic cells, monocytes, and progenitors. *Science* **356**, eaah4573 (2017).
10. Emoto, T. *et al.* Single-Cell RNA Sequencing Reveals a Distinct Immune Landscape of Myeloid Cells in Coronary Culprit Plaques Causing Acute Coronary Syndrome. *Circulation* **145**, 1434–1436 (2022).
11. The Immunological Genome Project Consortium, Dwyer, D. F., Barrett, N. A. & Austen, K. F. Expression profiling of constitutive mast cells reveals a unique identity within the

immune system. *Nat. Immunol.* **17**, 878–887 (2016).

12. Lagraauw, H. M., Wezel, A., Van Der Velden, D., Kuiper, J. & Bot, I. Stress-induced mast cell activation contributes to atherosclerotic plaque destabilization. *Sci. Rep.* **9**, 2134 (2019).

13. Winkels, H. & Wolf, D. Heterogeneity of T Cells in Atherosclerosis Defined by Single-Cell RNA-Sequencing and Cytometry by Time of Flight. *Arterioscler. Thromb. Vasc. Biol.* **41**, 549–563 (2021).

14. Fernandez, D. M. *et al.* Single-cell immune landscape of human atherosclerotic plaques. *Nat. Med.* **25**, 1576–1588 (2019).

15. Slysz, J. *et al.* Single-cell profiling reveals inflammatory polarization of human carotid versus femoral plaque leukocytes. *JCI Insight* **8**, e171359 (2023).

16. Kuret, T. & Sodin-Šemrl, S. The Role of Fibroblasts in Atherosclerosis Progression. in *Biochemistry* (eds. Frank Bertoncelj, M. & Lakota, K.) vol. 25 (IntechOpen, 2021).

17. Dib, L. *et al.* Lipid-associated macrophages transition to an inflammatory state in human atherosclerosis, increasing the risk of cerebrovascular complications. *Nat. Cardiovasc. Res.* **2**, 656–672 (2023).

18. Bonaccorsi, I. *et al.* Symptomatic Carotid Atherosclerotic Plaques Are Associated With Increased Infiltration of Natural Killer (NK) Cells and Higher Serum Levels of NK Activating Receptor Ligands. *Front. Immunol.* **10**, 1503 (2019).

19. Tamargo, I. A., Baek, K. I., Kim, Y., Park, C. & Jo, H. Flow-induced reprogramming of endothelial cells in atherosclerosis. *Nat. Rev. Cardiol.* **20**, 738–753 (2023).

20. Jiang, Y. *et al.* Endothelial Aquaporin-1 (AQP1) Expression Is Regulated by Transcription Factor Mef2c. *Mol. Cells* **39**, 292–298 (2016).

21. Elmasri, H. *et al.* Endothelial cell-fatty acid binding protein 4 promotes angiogenesis: role of stem cell factor/c-kit pathway. *Angiogenesis* **15**, 457–468 (2012).

22. Theodorou, K. & Boon, R. A. Endothelial Cell Metabolism in Atherosclerosis. *Front. Cell Dev. Biol.* **6**, 82 (2018).

23. Slenders, L. *et al.* Identification of endothelial-to-mesenchymal transition gene signatures in single-cell transcriptomics of human atherosclerotic tissue. Preprint at https://doi.org/10.1101/2023.07.18.549599 (2023).

24. Zhu, L. *et al.* Mutations in myosin heavy chain 11 cause a syndrome associating thoracic aortic aneurysm/aortic dissection and patent ductus arteriosus. *Nat. Genet.* **38**, 343–349 (2006).

25. Jin, S. *et al.* Notch Signaling Regulates Platelet-Derived Growth Factor Receptor-β Expression in Vascular Smooth Muscle Cells. *Circ. Res.* **102**, 1483–1491 (2008).

26. Schlosser, A. *et al.* MFAP4 Promotes Vascular Smooth Muscle Migration, Proliferation and Accelerates Neointima Formation. *Arterioscler. Thromb. Vasc. Biol.* **36**, 122–133 (2016).

27. Grootaert, M. O. J. & Bennett, M. R. Vascular smooth muscle cells in atherosclerosis: time for a re-assessment. *Cardiovasc. Res.* **117**, 2326–2339 (2021).

28. Wirka, R. C. *et al.* Atheroprotective roles of smooth muscle cell phenotypic modulation and the TCF21 disease gene as revealed by single-cell analysis. *Nat. Med.* **25**, 1280–1289 (2019).

29. Boltjes, A. & Van Wijk, F. Human Dendritic Cell Functional Specialization in Steady-State and Inflammation. *Front. Immunol.* **5**, (2014).

30. Britsch, S., Langer, H., Duerschmied, D. & Becher, T. The Evolving Role of Dendritic Cells in Atherosclerosis. *Int. J. Mol. Sci.* **25**, 2450 (2024).

31. Ghanem, M. H. *et al.* Proteomic and Single-Cell Transcriptomic Dissection of Human Plasmacytoid Dendritic Cell Response to Influenza Virus. *Front. Immunol.* **13**, 814627 (2022).

32. Chistiakov, D. A., Orekhov, A. N., Sobenin, I. A. & Bobryshev, Y. V. Plasmacytoid dendritic cells: development, functions, and role in atherosclerotic inflammation. *Front. Physiol.* **5**, (2014).

33. Siakaeva, E. *et al.* Neutrophil Maturation and Survival Is Controlled by IFN-Dependent Regulation of NAMPT Signaling. *Int. J. Mol. Sci.* **20**, 5584 (2019).

34. Shaath, H., Vishnubalaji, R., Elkord, E. & Alajez, N. M. Single-Cell Transcriptome Analysis Highlights a Role for Neutrophils and Inflammatory Macrophages in the Pathogenesis of Severe COVID-19. *Cells* **9**, 2374 (2020).

35. Kapellos, T. S. *et al.* Systemic alterations in neutrophils and their precursors in early-stage chronic obstructive pulmonary disease. *Cell Rep.* **42**, 112525 (2023).

36. Hong, Y. *et al.* Single-cell transcriptome profiling reveals heterogeneous neutrophils with prognostic values in sepsis. *iScience* **25**, 105301 (2022).

37. Kirchberger, S. *et al.* Comparative transcriptomics coupled to developmental grading via transgenic zebrafish reporter strains identifies conserved features in neutrophil maturation. *Nat. Commun.* **15**, 1792 (2024).

38. Moniaga, C. S., Watanabe, S., Honda, T., Nielsen, S. & Hara-Chikuma, M. Aquaporin-9-expressing neutrophils are required for the establishment of contact hypersensitivity. *Sci. Rep.* **5**, 15319 (2015).

39. Zhang, X., Kang, Z., Yin, D. & Gao, J. Role of neutrophils in different stages of atherosclerosis. *Innate Immun.* **29**, 97–109 (2023).

40. Cochain, C. & Zernecke, A. Macrophages in vascular inflammation and atherosclerosis. *Pflüg. Arch. - Eur. J. Physiol.* **469**, 485–499 (2017).

41. Lin, J.-D. *et al.* Single-cell analysis of fate-mapped macrophages reveals heterogeneity, including stem-like properties, during atherosclerosis progression and regression. *JCI Insight* **4**, e124574 (2019).

42. Wieland, E. B., Kempen, L. J., Donners, M. M., Biessen, E. A. & Goossens, P. Macrophage heterogeneity in atherosclerosis: A matter of context. *Eur. J. Immunol.* **54**, 2350464 (2024).

43. Zernecke, A. *et al.* Integrated single-cell analysis-based classification of vascular mononuclear phagocytes in mouse and human atherosclerosis. *Cardiovasc. Res.* **119**, 1676–1689 (2023).

44. Ramachandran, P. *et al.* Resolving the fibrotic niche of human liver cirrhosis at single-cell level. *Nature* **575**, 512–518 (2019).

45. Jaitin, D. A. *et al.* Lipid-Associated Macrophages Control Metabolic Homeostasis in a Trem2-Dependent Manner. *Cell* **178**, 686-698.e14 (2019).

46. Yu, L. *et al.* Heterogeneity of macrophages in atherosclerosis revealed by single‐cell RNA sequencing. *FASEB J.* **37**, e22810 (2023).
