# Supplementary material for: Integrated single-cell atlas of human atherosclerotic plaques
Source: Nat Commun. 2025 Sep 10;16:8255. doi: 10.1038/s41467-025-63202-x (PMC12423310; doi:10.1038/s41467-025-63202-x)
Supplement: Supplementary file 2 — Description of Additional Supplementary Files [file 41467_2025_63202_MOESM2_ESM.pdf]

Description of Additional Supplementary Files

File name: Supplementary Data 1
Description: Expression matrix from bulk RNA-seq profiling of 201 carotid artery samples. Preprocessing used fastp v0.23.2 for adapter clipping and quality trimming, and Salmon v1.6.0 for transcript quantification against GENCODE v40 (GRCh38). Rows list genes and columns list samples; the leading row encodes lesion stage (early vs. late).
